# Analgesic Effect of Ropivacaine Combined with Hydromorphone following Surgery for Mixed Hemorrhoids: A Pilot Study

**DOI:** 10.1155/2022/2033580

**Published:** 2022-02-04

**Authors:** Xuejing Luo, Yanfei Xia, Mengting Gu, Jin Yao

**Affiliations:** ^1^Department of Anesthesiology, Zhejiang Hospital, Hangzhou, China; ^2^Department of Anorectal Medicine, Zhejiang Hospital, Hangzhou, China

## Abstract

**Background:**

Postoperative pain is a major adverse effect of surgery for mixed hemorrhoids. We evaluated whether spinal anesthesia with ropivacaine and hydromorphone provided safe and effective analgesia after surgery for mixed hemorrhoids.

**Methods:**

This single-center, double-blind pilot study included patients with mixed hemorrhoids who underwent a procedure for prolapse and hemorrhoids (PPH) and external hemorrhoidectomy under spinal anesthesia at Zhejiang Hospital, China (October 2020 to December 2020). Patients were randomized to a hydromorphone group (spinal anesthesia with 0.5% ropivacaine and 75 *μ*g hydromorphone) or morphine group (spinal anesthesia with 0.5% ropivacaine and 150 *μ*g morphine). Pain scores (numerical rating scale), incidences of vomiting and itching, and length of hospital stay (LoS) were recorded at 6, 12, 18, and 24 hours after surgery.

**Results:**

The analysis included 40 patients in each group. Median (interquartile range) pain score in the hydromorphone group was higher than that in the morphine group at 12 hours (1 (0–2] vs. 0 (0–2), *p*=0.044) but not significantly different between groups at 6 hours (0 (0–1) vs. 0 (0-0) *p*=0.228), 18 hours (2 (2–3) vs. 2 (1–3) *p*=0.060) or 24 hours (2 (2–3) vs. 2 (1–3) *p*=0.081). The hydromorphone group had a lower incidence of pruritus than the morphine group (47.5% vs. 67.5%, *p*=0.018). There were no significant differences between groups in vomiting incidence or LoS.

**Conclusion:**

In patients with mixed hemorrhoids, spinal anesthesia with ropivacaine/hydromorphone has a comparable analgesic effect and a lower incidence of pruritus during the first 24 hours after surgery than spinal anesthesia with ropivacaine/morphine.

## 1. Introduction

Hemorrhoid disease is very common, affecting 11% of people worldwide [1]. Patients with mixed hemorrhoids have both internal hemorrhoids (which lie above the dentate line and show varying degrees of prolapse) and external hemorrhoids (which are located below the dentate line and can undergo thrombosis) [2]. Hemorrhoids are associated with symptoms such as pain, bleeding, and pruritus; hence, many patients seek treatment for this disorder [2]. The management of hemorrhoid disease includes conservative strategies such as lifestyle modification, fiber supplementation, anti-inflammatory drugs, venotonic drugs, sclerotherapy and rubber band ligation, and surgical interventions such as hemorrhoidectomy and stapled hemorrhoidopexy [3]. Surgery is the main method used to treat mixed hemorrhoids [4]. However, the anorectal and perianal regions are sensitive areas, and most perianal operations, including surgery for mixed hemorrhoids, cause severe postoperative pain [3, 5–7].

Clinical practice guidelines recommend that patients undergoing anorectal surgery receive multimodal analgesia, including oral or intravenous opioids, nonsteroidal anti-inflammatory drugs, and paracetamol [8, 9]. However, many patients still experience substantial postoperative pain after anorectal surgery despite the use of multimodal analgesia [10]. Spinal anesthesia with morphine and local anesthetics is often used as a component of multimodal analgesia for lower abdominal surgery such as cesarean section and prostatectomy [11, 12]. Furthermore, the addition of morphine to a local anesthetic drug during spinal anesthesia has been shown to improve short-term postoperative analgesia in patients undergoing hemorrhoidectomy [13, 14]. However, intrathecal administration of morphine is associated with numerous adverse effects such as vomiting and pruritus [12, 15].

Hydromorphone is an opioid drug that has been shown to reduce postoperative pain after knee surgery when administered intrathecally with a local anesthetic [16]. Furthermore, the analgesic effects of intrathecal hydromorphone after cesarean section are comparable to those of morphine [17, 18]. Hydromorphone has a similar molecular structure to morphine but is more soluble in lipids [19]. The higher lipid solubility of hydromorphone may reduce the incidence of adverse effects in patients compared with morphine [20]. However, few studies have compared the analgesic and adverse effects between hydromorphone and morphine when these drugs are administered intrathecally with a local anesthetic after surgery for mixed hemorrhoids.

This study aimed to compare the postoperative analgesia and adverse effects of hydromorphone and morphine when each drug was administered with ropivacaine as spinal anesthesia following surgical management of mixed hemorrhoids. It was anticipated that the study findings would provide useful information to help clinicians select appropriate management strategies to reduce postoperative pain in patients with mixed hemorrhoids.

## 2. Materials and Methods

### 2.1. Study Design

This study is a double-blind, parallel-group, randomized pilot study conducted at Zhejiang Hospital, Zhejiang, China. Written informed consent was obtained from each patient. This study is approved by Zhejiang Hospital Ethics Review Committee (2020 clinical trial no. (73K)) and is registered at the China Clinical Trial Registration Center (registration no. ChiCTR2000038457).

### 2.2. Patients

Patients with mixed hemorrhoids scheduled to undergo a procedure for prolapse and hemorrhoids (PPH) and external hemorrhoidectomy at Zhejiang Hospital between October 2020 and December 2020 were enrolled consecutively. The inclusion criteria were 18–60 years old, diagnosed with mixed hemorrhoids, American Association of Anesthesiologists (ASA) class I or II, and scheduled for PPH and external hemorrhoidectomy under spinal anesthesia. The exclusion criteria included the following: history of opioid abuse, long-term use of opioids due to chronic pain, and failure of lumbar anesthesia necessitating a switch to another method of anesthesia. The withdrawal criteria were not followed up after surgery, and a patient withdrew his consent.

### 2.3. Randomization and Blinding

Patients were randomized to a hydromorphone group (spinal anesthesia with ropivacaine and hydromorphone) or morphine group (spinal anesthesia with ropivacaine and morphine) using a random number table method. The random number table was created by a statistician and used by the pharmacist to dispense the appropriate drug to the anesthesiologist present at the operation. The patients, anorectal surgeons, nurses, and investigators were blinded to the grouping.

In order to determine the group to which each case belonged (group 1 or group 2), the data were partially unblinded after being collected and entered into the analysis software. Full unblinding to establish the actual grouping (group 1 = hydromorphone and group 2 = morphine) was only carried out after the data analysis had been completed.

### 2.4. Surgical Procedures

Peripheral vein access was obtained, and Ringer's lactate solution was infused intravenously at a rate of 5 mL/min. Noninvasive blood pressure, oxygen saturation, and electrocardiogram were routinely monitored. The L3-L4 space was selected for subarachnoid puncture with the patient in the lateral position. After subarachnoid puncture had been successfully achieved, the patient was administered 0.5% ropivacaine together with either 75 *μ*g hydromorphone (hydromorphone group) or 150 *μ*g morphine (morphine group) at an injection rate of 0.2 mL/s (total volume administered, 3 mL). Ephedrine was given as needed during the operation to keep the blood pressure fluctuations within 20% of the baseline blood pressure. Atropine was administered if the patient's heart rate fell below 50 beats/min.

After anesthesia had been achieved, the patient was placed in the prone position. The anus was expanded to the width of four fingers, and an anal dilator was inserted. A purse-string suture was placed 2 cm above the dentate line using 2/0 absorbable thread, and an anorectal stapler was then inserted and fired to remove excess hemorrhoidal tissue. Any areas of bleeding were managed using absorbable sutures. External hemorrhoids were treated by external stripping and internal ligation, and any bleeding was stopped by electrocoagulation under direct vision.

All patients were given parecoxib 40 mg b.i.d. for postoperative analgesia. Tropisetron (5 mg) was administered intravenously as needed to prevent postoperative vomiting. Pruritus was treated with intravenous nalbuphine infusion (5 mg every 4 hours) as needed. Tramadol was given by intravenous injection according to the pain score: < 4, none; 4–6, 50 mg; and 7–10, 100 mg.

### 2.5. Data Collection

The following demographic characteristics, baseline clinical characteristics, and operative characteristics were recorded for each patient: sex, age, height, weight, body mass index (BMI, defined as weight in kilograms divided by the square of height in meters) [21], and duration of surgery (defined as the interval between the time that anesthesia was completed and the time that skin suturing was completed).

On the day before surgery, preoperative visits and evaluations were conducted, informed consents for anesthesia and the trial were signed, and baseline characteristics (sex, age, height, weight, and body mass index) were collected. The patients were followed up every 6 h postoperatively to collect pain scores, opioid use, vomiting, and itching. The data were collected at the end of the 24 h postoperative follow-up, including the duration of surgery, pain scores at 6, 12, 18, and 24 h postoperatively, the number and dose of opioid use, and the frequency of vomiting and pruritus. At the beginning of each month of the trial, the patients' length of stay in the previous month was collected until all the patients in the trial were discharged.

### 2.6. Outcomes

Pain intensity was evaluated using the 11-point numerical rating scale (NRS) score, which ranges from 0 (no pain) to 10 (severe pain) [22]. The main outcome measure was pain score 24 hours after surgery. The secondary outcome measures were pain scores at 6 hours, 12 hours, and 18 hours after surgery, opioid use within 24 hours after surgery, incidences of vomiting and pruritus, and length of hospital stay (LoS).

### 2.7. Statistical Analysis

SPSS 25.0 (IBM, Armonk, NY, USA) was used for data analysis. Continuous variables are expressed as mean ± standard deviation (normal distribution) or median and interquartile range (non-normal distribution), and categorical variables are expressed as number and percentage. The independent *t*-test was used to compare baseline characteristics and LoS, and the nonparametric Wilcoxon rank-sum test was used to compare pain scores between the two groups. The chi-squared test or Fisher's exact probability test was used to compare opioid use and opioid side effects between the two groups. *p* < 0.05 was considered statistically significant.

## 3. Results

### 3.1. Demographic and Baseline Clinical Characteristics of the Patients

**Figure 1:**
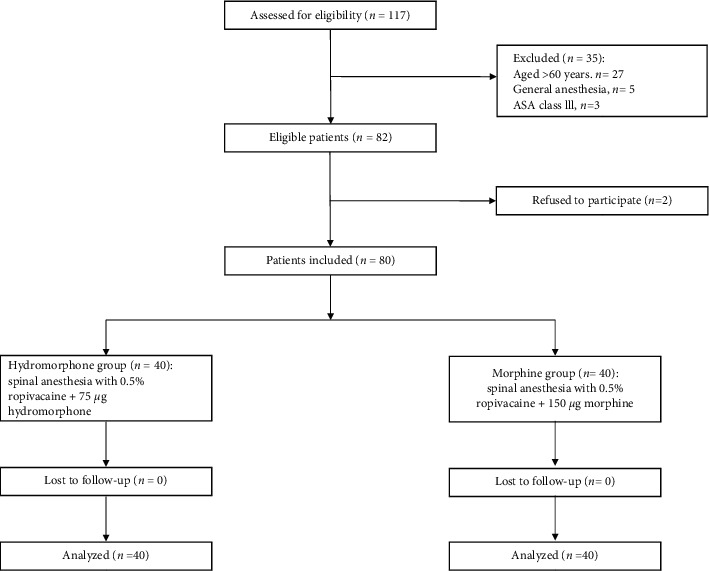
Recruitment of study participants. ASA: American Association of Anesthesiologists.

A total of 80 patients were randomized to the study groups (*n* = 40 for each group). All 80 patients completed the study and were included in the final analysis (Figure 1). The patients' demographic and baseline clinical characteristics are presented in Table 1. There were no significant differences between the hydromorphone and morphine groups in sex, age, height, weight, BMI, or duration of surgery (Table 1). During the study, no patients refused follow-up or asked to withdraw from the trial.

### 3.2. Postoperative Pain Scores

The postoperative pain scores are summarized in Table 2. The NRS pain score at 24 hours did not differ significantly between the hydromorphone group and morphine group (2 (2–3) vs. 2 (1–3), *p*=0.081; Table 2). Although the pain score was higher in the hydromorphone group than in the morphine group at 12 hours (1 (0–2) vs. 0 (0–2), *p*=0.044), it was not significantly different between groups at 6 hours (0 (0–1) vs. 0 (0–0), *p*=0.228) or 18 hours (2 (2–3) vs. 2 (1–3), *p*=0.060; Table 2).

### 3.3. Safety and Adverse Effects

The safety data are summarized in Table 3. No patients in either group had a pain score higher than 7. The number of patients requiring an intravenous injection of 50 mg tramadol did not differ significantly between the hydromorphone group (*n* = 3, 7.5%) and morphine group (*n* = 2, 5%). There was no significant difference between groups in the incidence of vomiting or the proportion of patients administered tropisetron (Table 3). Notably, the number of patients with pruritus was significantly lower (*p*=0.018) in the hydromorphone group (*n* = 19, 47.5%) than in the morphine group (*n* = 27, 67.5%). There was also a trend toward less frequent use of nalbuphine in the hydromorphone group (*n* = 8, 20.0%) than in the morphine group (*n* = 15, 37.5%), although statistical significance was not attained (*p*=0.084). Additionally, there was no significant difference in length of hospital stay between the hydromorphone group (6.7 ± 2.1 days) and the morphine group (6.8 ± 1.7 days).

## 4. Discussion

Inadequate postoperative analgesia after surgery for mixed hemorrhoids will limit patients' mobility and self-care ability and reduce their quality of life. An important finding of the present study was that spinal anesthesia with hydromorphone and ropivacaine provided a comparable analgesic effect to spinal anesthesia with morphine and ropivacaine, with both methods achieving a good level of analgesia according to the NRS pain scores. Furthermore, spinal anesthesia with hydromorphone and ropivacaine was associated with a lower incidence of pruritus than spinal anesthesia with morphine and ropivacaine, while the incidence of vomiting was similar between groups. Our findings indicate that hydromorphone is an acceptable alternative to morphine for use in spinal anesthesia with ropivacaine and may have the advantage of a lower incidence of pruritus.

Spinal anesthesia that combines morphine with a local anesthetic is widely used as part of multimodal analgesia and has been shown to exert a good analgesic effect [11–14]. Moreover, numerous studies have reported that the addition of morphine to a local anesthetic improves postoperative analgesia in patients undergoing anorectal surgery, including hemorrhoidectomy [13, 14, 23, 24]. Although previous clinical research has indicated that intrathecal hydromorphone produces comparable analgesic effects to intrathecal morphine in women undergoing cesarean section [17, 18], no previous investigations have compared the postoperative analgesic effects of hydromorphone and morphine after surgery for mixed hemorrhoids. In the present study, we added either 75 *μ*g hydromorphone or 150 *μ*g morphine to the ropivacaine solution used for spinal anesthesia. These drug concentrations were selected according to the 90% effective doses determined by Sviggum et al. based on the pain score 12 hours after administration [25]. Importantly, we found that the pain scores at 6 hours, 18 hours, and 24 hours were not significantly different between the hydromorphone group and morphine group, which agrees with previous studies reporting similar analgesic effects for these two opioids [17, 18, 26].

Opioids are associated with various adverse effects, including nausea, vomiting, and pruritus [12, 15]. Some previous investigations have reported that the incidence of side effects and LoS were similar between hydromorphone and morphine [17, 18, 25, 26]. Consistent with this previous research, we also observed no significant differences in the incidence of vomiting or LoS between the hydromorphone group and morphine group. However, it was notable that the hydromorphone group had a significantly lower incidence of pruritus than the morphine group. The abovementioned finding is consistent with a prior study of children undergoing orthopedic surgery, which showed that pruritus was more severe and frequent for epidural morphine than for epidural hydromorphone. The pathophysiological mechanism by which morphine in the spinal canal causes itching is unclear but may involve activation of *μ* opioid receptors, 5-hydroxytryptamine (5-HT) receptors, and the itching center in the central nervous system as well as interactions between itching and pain. There are abundant 5-HT_3_ receptors in the spinal dorsal horn and trigeminal spinal tract, and these receptors are often coexpressed with opioid receptors and regulate pain conduction and gastrointestinal motility. Morphine can cause skin itching by activating 5-HT_3_ receptors, and some studies have shown that 5-HT_3_ antagonists can prevent itching caused by morphine in the spinal canal [27, 28]. Furthermore, the incidence and severity of itching increase as the intrathecal dose of morphine is increased [29, 30]. Hydromorphone and morphine have similar molecular structures, but hydromorphone is more lipid-soluble. We speculate that the cerebrospinal fluid concentration of lipophilic hydromorphone decreases faster than that of hydrophilic morphine, resulting in a lower incidence of pruritus for intrathecal hydromorphone than for intrathecal morphine.

## 5. Conclusions

In patients with mixed hemorrhoids, spinal anesthesia using hydromorphone and ropivacaine produced an analgesic effect during the first 24 hours after surgery compared to that of spinal anesthesia using morphine and ropivacaine. In addition, the incidence of pruritus was lower in patients administered hydromorphone than in patients administered morphine. Our results suggest that hydromorphone is an acceptable alternative to morphine for use with ropivacaine in spinal anesthesia and that hydromorphone may have the advantage of a lower incidence of pruritus. Large-scale, multicenter studies are needed to confirm our findings.

### 5.1. Limitations

This study has some limitations. First, this is a single-center study, so whether the results are generalizable remains unknown. Second, the sample size is quite small, so the study may have been underpowered to detect some real differences between groups. This study is exploratory, and no sample size calculation was performed. A randomized controlled trial is currently being designed to confirm the results. Third, we utilized a multimodal analgesia strategy after surgery, which included the administration of parecoxib to all patients and intravenous administration of tramadol as needed. Although these treatments are suitable for clinical practice, their use may have masked differences in pain scores between groups. Fourth, NRS pain scores were recorded at only four time points during the first 24 hours after surgery, so it remains possible that the analgesic effects of hydromorphone and morphine may have differed at other time points during or after the initial 24-hour period.

## Figures and Tables

**Table 1:** Demographic and baseline clinical characteristics of the study participants.

Characteristic	Hydromorphone group (*n* = 40)	Morphine group (*n* = 40)	*t*-value	*p* value
Male, *n* (%)	25 (62.5%)	22 (55.0%)		
Age (years), mean ± SD	39.7 ± 10.7	39.5 ± 10.8	0.084	0.933
Height (cm), mean ± SD	167.5 ± 8.6	167.1 ± 8.2	0.213	0.832
Weight (kg), mean ± SD	68.2 ± 14.1	63.8 ± 10.9	1.539	0.128
Body mass index (kg/m^2^), mean ± SD	24.1 ± 3.8	22.8 ± 3.0	1.789	0.077
Duration of surgery (min), mean ± SD	27.4 ± 10.0	25.4 ± 8.0	0.964	0.338

SD: standard deviation.

**Table 2:** Numerical rating scale (NRS) pain scores.

Time post operation (h)	Hydromorphone group (*n* = 40)	Morphine group (*n* = 40)	*Z*-value	*p* value
6	0 (0–1)	0 (0–0)	−1.198	0.228
12	1 (0–2)	0 (0–2)	−2.056	0.044
18	2 (1–3)	2 (0–2)	−1.891	0.060
24	2 (2–3)	2 (1–3)	−1.751	0.081

Data are expressed as the median (interquartile range). The Wilcoxon rank-sum test was used for statistical comparisons between groups.

**Table 3:** Other outcomes.

	Hydromorphone group (*n* = 40)	Morphine group (*n* = 40)	*χ* ^2^ */t*-value	*p* value
Tramadol, *n* (%)	3 (7.5%)	2 (5.0%)	0.213	>0.999
Tropisetron, *n* (%)	3 (7.5%)	2 (5.0%)	0.213	>0.999
Nalbuphine, *n* (%)	8 (20.0%)	15 (37.5%)	2.990	0.084
Vomiting, *n* (%)	7 (17.5%)	6 (15.0%)	0.092	>0.999
Pruritus, *n* (%)	19 (47.5%)	27 (67.5%)	3.274	0.018
LoS (days), mean ± SD	6.7 ± 2.1	6.8 ± 1.7	−0.178	0.271

LoS: length of hospital stay; SD: standard deviation.

## Data Availability

The data can be obtained from the corresponding author upon reasonable request.

## Supplementary Materials

Supplementary Materials CONSORT 2010 checklist.
